# Developing a multiplex loop-mediated isothermal amplification assay (LAMP) to determine severe fever with thrombocytopenia syndrome (SFTS) and scrub typhus

**DOI:** 10.1371/journal.pone.0262302

**Published:** 2022-02-16

**Authors:** Woong Sik Jang, Da Hye Lim, Young Lan Choe, Jeonghun Nam, Kyung Chul Moon, Chaewon Kim, Minkyeong Choi, Insu Park, Dae Won Park, Chae Seung Lim

**Affiliations:** 1 Emergency Medicine, College of Medicine, Korea University Guro Hospital, Seoul, Korea; 2 Departments of Laboratory Medicine, College of Medicine, Korea University Guro Hospital, Seoul, Korea; 3 Department of Song‐do Bio Engineering, Incheon Jaeneung University, Incheon, Korea; 4 Artificial Intelligence (AI)‐Bio Research Center, Incheon Jaeneung University, Incheon, Korea; 5 Division of Infectious Diseases, Department of Internal Medicine, Korea University Ansan Hospital, Ansan-si, Gyeonggi-do, Republic of Korea; New England Biolabs Inc, UNITED STATES

## Abstract

Severe fever with thrombocytopenia syndrome (SFTS) and scrub typhus are endemic zoonotic diseases that pose significant public health threats in East Asia. As these two diseases share common clinical features, as well as overlapping disease regions, it is difficult to differentiate between SFTS and scrub typhus. A multiplex reverse-transcription loop‑mediated isothermal amplification (RT-LAMP) assay was developed to detect large segments and GroES genes for SFTS virus (SFTSV) and *Orientia tsutsugamushi* (OT). The performance of the RT-LAMP assay was compared and evaluated with those of commercial PowerChek^™^ SFTSV real-time PCR and LiliF^™^ TSUTSU nested PCR for 23 SFTS and 12 scrub typhus clinical samples, respectively. The multiplex SFTSV/OT/Internal control (IC) RT-LAMP assay showed comparable sensitivity (91.3%) with that of commercial PowerChek^™^ SFTSV Real-time PCR (95.6%) and higher sensitivity (91.6%) than that of LiliF^™^ TSUTSU nested PCR (75%). In addition, the multiplex SFTSV/OT RT-LAMP assay showed 100% specificity and no cross-reactivity for blood from uninfected healthy patients and samples from patients infected with other fever viruses. Thus, the multiplex SFTSV/OT/IC RT-LAMP assay could serve as a useful point-of-care molecular diagnostic test for SFTS and scrub typhus.

## Introduction

Severe fever with thrombocytopenia syndrome (SFTS) and scrub typhus are endemic zoonotic diseases that are becoming significant public health threats in East Asia [1, 2]. The causative pathogen of SFTS is severe fever thrombocytopenia syndrome virus (SFTSV), which is a newly identified pathogenic member of the Phlebovirus species in the family Bunyaviridae [3]. The SFTSV is usually transmitted by ticks such *as Haemophysalis longicornis* during outdoor activities [4]. SFTS has been mainly reported in East Asian countries, including Korea, China, and Japan, with a high mortality rate (6.3–30%) [5–7]. The clinical features of SFTS are fever, headache, myalgia, and gastrointestinal symptoms, followed by thrombocytopenia and leukopenia [8].

Scrub typhus is caused by *Orientia tsutsugamushi* (OT), an obligate intracellular bacterium mediated by chigger mites such as *Leptotrombidium* species [9]. The distribution of scrub typhus is thought to be endemic to the region known as the “tsutsugamushi triangle,” which includes China, Japan, Indonesia, Malaysia, Thailand, Pakistan, Korea, northern Australia, and the islands of the western Pacific and Indian Ocean [10–12]. The mortality rate of scrub typhus is 1.4% [13], and the clinical course of scrub typhus is usually mild and self-limiting; however, delaying the treatment in severe cases can lead to complications such as renal failure, myocarditis, meningoencephalitis, and death [14–16]. Unfortunately, scrub typhus is characterized by symptoms such as fever, headache, myalgia, cough, and abdominal pain, which are difficult to differentiate from the symptoms of SFTS [8, 17]. Therefore, an incorrect diagnosis of patients with these symptoms followed by inadequate treatment can lead to death [18]. Therefore, it is necessary to differentiate and diagnose SFTS and Scrub typhus in the early stages [19].

Currently, the definitive diagnosis of scrub typhus and SFTS depends on laboratory-based diagnostic methods, including indirect immunofluorescent antibody test (IFA),​ enzyme-linked immunosorbent assay, immunochromatographic tests, and polymerase chain reaction (PCR) [20–23]. The current diagnostic criterion for scrub typhus is IFA testing [24]. However, single IFA measurements are sometimes insufficient for definitive diagnosis [12]. Recently, PCR has been developed and used for the early diagnosis of scrub typhus [25, 26]. For the diagnosis of SFTS, RT-PCR is mainly used but not standardized; therefore, it is difficult to diagnose SFTS quickly [27, 28]. In addition, since PCR tests are difficult to perform at regional hospitals due to lack of adequate resources, samples obtained from patients suspected of SFTS are mostly sent to the national laboratory, resulting in significant delays in PCR test results. Recently, a simple version of a practical molecular assay, the loop-mediated isothermal amplification (LAMP) assay was developed for use in resource-poor environments [29, 30]. This LAMP assay can be performed using simple equipment at a constant temperature, making it a fast and cost-effective point-of-care diagnostic test [31, 32]. In addition, inexpensive multi-channel isothermal amplifiers, such as Genie III (OptiGene, Horsham, UK) and T8-ISO (TwistDX, Cambridge, UK), have been developed and commercialized.

In this study, we developed a multiplex SFTSV/OT/IC loop‑mediated isothermal amplification (LAMP) assay for the differential diagnosis of SFTSV and *O*. *tsutsugamushi* infection, based on a slightly modified DARQ probe method [33, 34]. The detection limit of the multiplex SFTSV/OT/IC RT-LAMP assay was confirmed using diluted SFTS and scrub typhus clinical samples. The sensitivity and specificity of the RT-LAMP assay were compared and evaluated with those of the commercial PowerChek^™^ SFTSV Real-time PCR kit and LiliF^™^ TSUTSU nested PCR kit for SFTS and scrub typhus clinical samples, respectively.

## Materials and methods

### Clinical samples and nucleic acid extraction

We collected 35 clinical samples of serum and blood from 19 patients suspected of being infected with SFTSV (23 from 7 patients) and *O*. *tsutsugamushi* (12 from 12 patients) in the Republic of Korea. SFTS blood/serum samples (23) were collected on different days from hospitalized 7 SFTS patients. SFTS (23) and scrub typhus (12) clinical samples were confirmed using the SFTSV qRT-PCR and *O*. *tsutsugamushi* qPCR, which were previously reported by Yoshikawa *et*. *al*. [35] and Tantibhedhyangkul *et*. *al*. [26], respectively. To assess the specificity of the multiplex SFTSV/OT/IC RT-LAMP assay, 100 clinical sample specimens from individuals with (38) and without (62) other viral infections were tested. Viral infection samples, as confirmed via PCR using qRT-PCR [36–38] and the Anyplex^™^ II RV16 detection kit (Seegene, Seoul, South Korea), included 1 Hantaan virus, 4 Dengue virus (1–4), 1 Chikungunya virus, 4 influenza virus A/H1N1, 4 influenza virus A/H3N2, 4 influenza virus B, 4 respiratory syncytial virus (RSV) A, 4 RSV B, and 12 coronaviruses (KHU1, NL63, 229E). Nucleic acids (DNA and RNA), were extracted from 200 μL of clinical blood or serum samples using the cell DNA/RNA/NA kit (Genolution, Seoul, Korea) with Nextractor^®^ NX-48S (Genolution, Seoul, Korea), according to the manufacturer’s instructions. Nucleic acid samples were stored at -50 ºC before further testing. This study was approved by the Medical Ethics Committee of Korea University Guro Hospital (IRB No. 2020GR0556). Informed consent was waived by the institutional review board (IRB) because this study used residual samples.

### Primer design

The RT-LAMP primer sets for SFTSV and *O*. *tsutsugamushi* were designed from the conserved regions of the L segment and groES genes (Table 1). The actin beta gene in humans was used as an internal control (IC), as previously reported [39]. All LAMP primers including two outer primers (forward primer F3 and backward primer B3), two inner primers (forward inner primer FIP and backward inner primer BIP), and two loop primers (forward loop primer LF and backward loop primer LB) were designed using the Primer Explorer v4 software (Eiken Chemical Co., Tokyo, Japan). For multiple LAMPs, we used a slightly modified DARQ probe method. Briefly, a dye-labeled artificial nucleic acid + FLP sequence probe and a quencher-labeled displacement probe complementary to the artificial nucleic acid sequence were used. In this study, two types of artificial nucleic acids (32mers and 35mers) were used for multiplexing different fluorescence (FAM/Hex and Cy5) quenched by BHQ1 and BHQ2, respectively. A FAM (or Hex)-labeled 32-artificial oligomer-SFTS (or IC) FLP was designed for SFTS (or IC) FLP probe 1 and a Cy5-labeled 35-artificial oligomer-OT FLP was designed for tsu FLP probe 2. The quencher-labeled 30-oligonucleotide (BHQ1) or 35-oligonucleotide (BHQ2) was complementary to artificial nucleic acids sequences of SFTS (or IC) FLP probe 1 and tsu FLP probe 2, respectively. Before use in LAMP, all primers were assessed for specificity by performing a BLAST search. All LAMP primers and probes were synthesized by Macrogen (Seoul, South Korea).

**Table 1:** The multiplex SFTSV/OT/IC RT-LAMP primer sets.

Target	Name	Sequence (5´-3´)	Length (mer)	Conc of LAMP primer mix (20x)
SFTSV (L segment gene)	SFTSV F3	CCG ACT CAG GCT TTG GTT C	19	4 μM
	SFTSV B3	AAG GCA GGC TTG AAT CGC	18	4 μM
	SFTSV FIP	CTA CCC CCT CCA CAT TGC CYT TRA TGG ATC ATG GCG CAT A	40	32 μM
	SFTSV BIP	TCA GTG ACC CTG CAA AAG ARC TTT TGC CTC TTT GGG GGT RTC	42	32 μM
	SFTSV FLP	TTG CCC ACA GCT CAC CA	17	4 μM
	SFTSV BLP	CAT TGC YAT CTC TGA TGA TCC AGA W	25	10 μM
	SFTSV FLP probe1	FAM-CGG GCC CGT ACA AAG GGA ACA CCC ACA CTC CGT TGC CCA CAG CTC ACC A	49	6 μM
*O*. *tsutsugamushi* (GroES gene)	tsu F3	GAT TAT ATG AAA TAC CAA CCA CTG	24	4 μM
	tsu B3	TTT CAG TAC CAG CCC ATT	18	4 μM
	tsu FIP	CCT TTG CGG TAT CTG GAA TAA GAA TTA TGA TCG TGT GCT AGT TGA G	46	32 μM
	tsu BIP	CAG AGG GAA TAG TAG TTA TGG TTG GCC TTT CTT TAC TTT TAA CGG TGT A	49	32 μM
	tsu FLP	TTT ACC RTG TGC TTC ATC ATT GTG	24	4 μM
	tsu BLP	CGG GGG CTA TAG AAA TGA TAA AGG	24	10 μM
	tsu FLP probe2	CY5-GTC AGT GCA GGC TCC CGT GTT AGG ACG AGG GTA GGC GGG GGC TAT AGA AAT GAT AAA GG	59	6 μM
Internal control (Actin beta)	ACTB F3	AGT ACC CCA TCG AGC ACG	18	4 μM
	ACTB B3	AGC CTG GAT AGC AAC GTA CA	20	4 μM
	ACTB FIP	GAG CCA CAC GCA GCT CAT TGT ATC ACC AAC TGG GAC GAC A	40	32 μM
	ACTB BIP	CTG AAC CCC AAG GCC AAC CGG CTG GGG TGT TGA AGG TC	38	32 μM
	ACTB BLP	TGT GGT GCC AGA TTT TCT CCA	21	4 μM
	ACTB FLP	CGA GAA GAT GAC CCA GAT CAT GT	23	10 μM
	ACTB FLP probe1	HEX-CGG GCC CGT ACA AAG GGA ACA CCC ACA CTC CGC GAG AAG ATG ACC CAG ATC ATG T	55	6 μM
Quencher probe 1		GAG TGT GGG TGT TCC CTT TGT ACG GGC CCG-BHQ1	30	9 μM
Quencher probe 2		CCT ACC CTC GTC CTA ACA CGG GAG CCT GCA CTG AC-BHQ2	35	9 μM

### Real-time RT-PCR

To confirm SFTS and scrub typhus positive clinical samples, the SFTSV L, M, and S gene qRT-PCR primer sets [35] and *O*. *tsutsugamushi* groEL gene qPCR primer set [26] were used, and PCR conditions were set according to the protocol described by Yoshikawa *et al*., and Tantibhedhyangkul *et al*., respectively. The thermocycling parameters of the SFTSV L segment gene qRT-PCR were used as follows: after incubation at 95°C for 2 min, a reverse transcription step was performed at 55°C for 30 min, followed by 45 cycles of 94°C for 30 s, 52°C for 30 s with fluorescence detection, and 68°C for 30 s. The PCR cycling conditions for *O*. *tsutsugamushi* groEL gene qPCR were as follows: initial denaturation and hot-start enzyme activation at 95°C for 3 min, followed by 45 cycles of denaturation at 95°C for 1 s, combined annealing/extension at 58°C for 30 s with data acquisition for 12 s, and extension at 70°C for 3 s. PowerChek^™^ SFTSV Real-time PCR and LiliF^™^ TSUTSU nested PCR were carried out according to a manufacture’s protocol.

### Multiplex RT-LAMP

For multiplex SFTSV/OT/IC RT-LAMP assay, the reaction mixture was prepared with 12.5 μL of 2× reaction buffer, 1.25 μL of SFTSV LAMP primer mix (20x), 1.5 μL of OT LAMP primer mix (20x), 0.625 μL of IC (actin β) LAMP primer mix (20x), 1.875 μL of 9 μM quencher 1 solution for quenching the SFTS and IC LAMP probe, 1.5 μL of 9 μM quencher 2 solution for quenching the OT LAMP probe, and 5 μL of sample RNA (with a final reaction volume of 25 μL). The compositions of all LAMP primer mix (20x) were 4 μM of two outer primers (F3 and B3) and 32 μM of two inner primers (FIP and BIP), 10 μM of loop BLP primer, 4 μM loop FLP primer, and 6 μM loop FLP probe primer. The RT-LAMP assay was run on a CFX 96 Touch Real-Time PCR Detection System (Bio-Rad Laboratories, Hercules, CA, USA) at 62ºC for 40 min. The FAM, Hex, and Cy5 fluorescence channels were used to detect SFTSV, IC, and *O*. *tsutsugamushi*, respectively. For comparative studies, the performance of multiplex SFTSV/OT/IC LAMP assays was compared with that of commercial PowerChek^™^ SFTSV Real-time PCR kit and LiliF^™^ TSUTSU nested PCR Kit. The RT-LAMP assay was performed using the RT-LAMP 2× Master Mix (ELPIS-Biotech, Daejeon, South Korea).

### Limits of detection

pTOP Blunt V2 plasmids, including partial L segment gene sequences of SFTSV and groES gene sequences of *O*. *tsutsugamushi*, were used to test the limit of detection (LOD) of the RT-LAMP assay. All plasmids were constructed by Macrogen. To determine the LOD of the monoplex SFTSV RT-LAMP, monoplex OT RT-LAMP, and multiplex SFTSV/OT/IC RT-LAMP assays, the plasmids were serially diluted 10-fold from 1 × 10^6^ to 1 × 10^0^ copies/μL. This test was repeated 3 times. In addition, the LODs of the monoplex SFTSV RT-LAMP, monoplex OT RT-LAMP, and multiplex SFTSV/OT/IC RT-LAMP assays were compared with those of the commercial PowerChek^™^ SFTSV Real-time PCR kit and LiliF^™^ TSUTSU nested PCR kit for 2-fold serial dilutions of clinical samples from patients infected with SFTSV or *O*. *tsutsugamushi*. According to FDA EUA guidelines for COVID-19 diagnostic tests, the LODs of the monoplex SFTSV RT-LAMP, monoplex OT RT-LAMP, and multiplex SFTSV/OT/IC RT-LAMP assays were determined as the minimum concentration in a 2-fold dilution series at which 19 of 20 replicates amplify [40, 41]. the LODs of commercial PowerChek^™^ SFTSV Real-time PCR kit and LiliF^™^ TSUTSU nested PCR kit were repeated five times and determined as the minimum concentration in a 2-fold dilution series at which 5 of 5 replicates amplify.

## Results

### Optimization of multiplex SFTSV/OT/IC RT-LAMP assay

**Fig 1:**
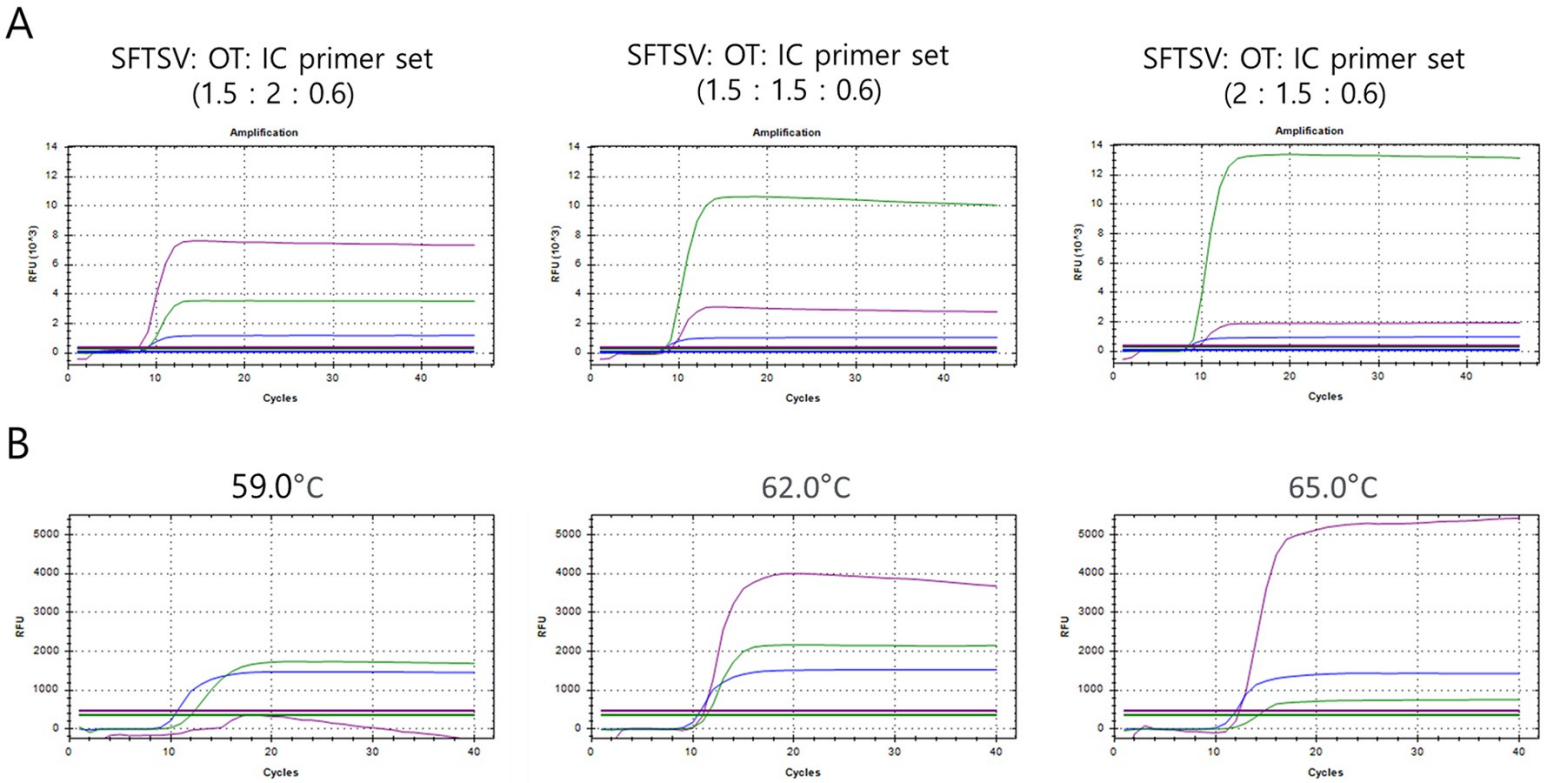
Optimization of the multiplex SFTSV/OT/IC LAMP primer set. (A) Different concentration ratios of SFTSV, OT, and IC primer sets (1.5:2:0.6, 1.5:1.5:0.6, and 2:1.5:0.6, respectively) for SFTSV, OT, and IC plasmid mixtures (1:1:1). (B) Temperature gradient tests (59–65°C) of the multiplex SFTSV/OT/IC LAMP assay.

The sensitivities of the monoplex SFTSV (FAM) and monoplex OT (Hex) RT-LAMP assay using strand-displaceable probes were evaluated by testing synthetic plasmid standards, including synthetic partial L segment gene of SFTSV and groES genes of *O*. *tsutsugamushi* ranging from 10^6^ to 10^0^ copies/μL, respectively. The limits of detection of monoplex SFTSV RT-LAMP and monoplex OT RT-LAMP were 1x10^2^ copies/μL and 1x10^1^ copies/μL, respectively (Table 2). For optimization of multiplex SFTSV /OT (Cy5)/IC (Hex) RT-LAMP assays, different ratios (1.5:2:0.6, 1.5:1.5:0.6 and 2:1.5:0.6) of LAMP primers set (vol, μL/ 25 μL reaction) were tested at 62ºC with synthetic plasmids including partial L segment gene of SFTSV and groES genes of *O*. *tsutsugamushi* (Fig 1A). For internal control (IC), actin beta LAMP primer set was used for the human actin beta gene amplification (Table 1). In SFTSV signal detection, a ratio of 1.5:1.5:0.6 showed faster Ct values (8.27) than others (1.5:2:0.6 = 8.94 and 2:1.5:0.6 = 8.30). In OT signal detection, a ratio of 1.5:2:0.6 showed faster Ct values (7.84) than others (1.5:1.5:0.6 = 9.18 and 2:1.5:0.6 = 9.59). Comprehensively considering the ct values and RFUs, the 1.5:2:0.6 ratio of the SFTS/OT/IC LAMP primer set was finally determined. Next, temperature-gradient tests (59, 62 and 65ºC) showed that the optimum temperature was 62ºC for multiplex SFTSV/OT/IC RT-LAMP assay (Fig 1B). Finally, in LOD test, the multiplex SFTSV/OT/IC RT-LAMP assay showed the same detection limit of monoplex SFTSV and OT RT-LAMP primer set for synthetic SFTSV and OT plasmid standards (Table 2).

**Table 2:** Limit of detection test for the monoplex and multiplex SFTSV/OT/IC LAMP primer set.

	**Plasmid**	**Monoplex RT-LAMP**		**Multiplex SFTSV/OT/IC RT-LAMP**					
		**SFTSV**		**SFTSV**		**IC**		***O*. *tsutsugamushi***	
		**Ct**	**RFU**	**Ct**	**RFU**	**Ct**	**RFU**	**Ct**	**RFU**
**SFTSV plasmid dilution sample (copies/ μL)**	**10** ^**6**^	**7.6**	**17581**	**8.5**	**21559**	N/A	71.7	N/A	-39.1
	**10** ^**5**^	**9.3**	**17842**	**10.5**	**20796**	N/A	75.8	N/A	241
	**10** ^**4**^	**10.1**	**17638**	**11.6**	**20939**	N/A	73.3	N/A	290
	**10** ^**3**^	**13.3**	**17760**	**12.7**	**21164**	N/A	66	N/A	318
	**10** ^**2**^	**17.5**	**18137**	**20.8**	**21961**	N/A	45.1	N/A	297
	**10** ^**1**^	**N/A**	**187**	**N/A**	**7**	N/A	-0.348	N/A	7.55
	**10** ^**0**^	**N/A**	**56**	**N/A**	**49**	N/A	3.61	N/A	-1.77
	**DW**	**N/A**	**134**	**N/A**	**97**	N/A	3.45	N/A	15.5
	**Plasmid**	***O*. *tsutsugamushi***		**SFTSV**		**IC**		***O*. *tsutsugamushi***	
		**Ct**	**RFU**	**Ct**	**RFU**	**Ct**	**RFU**	**Ct**	**RFU**
***O*. *tsutsugamushi* plasmid dilution sample (copies/ μL)**	**10** ^**6**^	**6.8**	**3939**	N/A	-8.32	N/A	-4.81	**8.1**	**5240**
	**10** ^**5**^	**7.9**	**4228**	N/A	-8.11	N/A	126	**9.4**	**5995**
	**10** ^**4**^	**9.7**	**4420**	N/A	-7.79	N/A	-7.44	**11.9**	**6079**
	**10** ^**3**^	**10.8**	**4351**	N/A	-1.47	N/A	-6.33	**11.8**	**6045**
	**10** ^**2**^	**11.9**	**4371**	N/A	-2.59	N/A	-6	**12.9**	**5782**
	**10** ^**1**^	**15**	**4081**	N/A	-10.1	N/A	-5.53	**15.2**	**5992**
	**10** ^**0**^	**18.4**	**4184**	N/A	0.334	N/A	2.33	**21.4**	**5921**
	**DW**	**N/A**	**42**	N/A	23.1	N/A	3.96	**N/A**	**25**

Each mean value of Ct and RFU is the average of the three LAMP assay repetitions.

### Comparison of detection limits of the multiplex SFTSV/OT/IC RT-LAMP assay with those of commercial kits for serial diluted SFTS and scrub typhus clinical samples

The LODs of the monoplex and multiplex SFTSV/OC/IC LAMP primer sets were compared to those of two commercial kits, the PowerChek^™^ SFTSV Real-time PCR kit (Kogene Biotech, Seoul, Korea) and LiliF^™^ TSUTSU nested PCR kit (iNtRON, Seongnam-Si, South Korea), for 2-fold serial diluted SFTSV serum samples and scrub typhus blood samples (range 2^8^–2^15^ and 2^1^−2^8^, respectively) (Table 3). For serial two-fold diluted SFTS clinical samples, monoplex and PowerChekTM SFTSV Real-time PCR kit showed the same LODs of 2^10^, whereas the LOD of multiplex SFTSV/OC/IC LAMP assay was 2^12^, which is one step lower than results of others. However, for scrub typhus clinical samples, both of the monoplex and multiplex SFTSV/OC/IC LAMP assay showed the superior LOD (2^5^) to that (2^3^) of the LiliFTM TSUTSU nested PCR kit.

**Table 3:** Limit of detection tests of the multiplex SFTSV/OT/IC RT-LAMP assay, PowerChek^™^ SFTSV Real-time PCR kit and LiliF^™^ TSUTSU nested PCR kit for two-fold diluted clinical samples from patients infected with SFTSV or *O*. *tsutsugamushi*.

Assays	Clinical samples (Nucleic acid extracted from clinical sample (2^n^) per reaction)															
	SFTSV								*O*. *tsutsugamushi*							
	8	9	10	11	12	13	14	15	1	2	3	4	5	6	7	8
**Monoplex SFTSV RT-LAMP**																
**Monoplex OT RT-LAMP**																
**Multiplex SFTSV/OT/IC RT-LAMP**																
**PowerChek**^**TM**^ **SFTSV Real-time PCR kit**																
**LiliF**^**TM**^ **TSUTSU nested PCR kit**																

### Comparison of the clinical performance of the multiplex SFTSV/OT/IC RT-LAMP assay with those of the commercial PowerChek^™^ SFTSV Real-time PCR kit and LiliF^™^ TSUTSU nested PCR kit using clinical samples

To confirm the clinical performance of the multiplex SFTSV/OT/IC RT-LAMP assay, the sensitivities of the assays were compared to those of the commercial PowerChek^™^ SFTSV Real-time PCR kit and LiliF^™^ TSUTSU nested PCR kit for 35 clinical samples from patients infected with SFTSV (23) and *O*. *tsutsugamushi* (12). For the specificities of these three assays, 100 clinical sample specimens from individuals with (38) and without (62) other viral infections were used (Table 4). For the SFTS clinical samples (n = 23), the sensitivity of the PowerChek^™^ SFTSV Real-time PCR kit was 95.6% (S gene: 95.6%, and M genes: 86.9%). The sensitivity of the multiplex SFTSV/OT/IC RT-LAMP assay was 91.3%. For scrub typhus clinical samples (n = 12), the sensitivity of the LiliF^™^ TSUTSU nested PCR kit was 75% and that of the multiplex SFTSV/OT/IC RT-LAMP assay was 91.6%. Overall, the multiplex SFTSV/OT/IC RT-LAMP assay showed similar sensitivities to that of the commercial PowerChek^™^ SFTSV Real-time PCR kit and superior sensitivity to the LiliF^™^ TSUTSU nested PCR kit. For 100 negative clinical samples (non-infection/other viral infections; n = 64/38), the specificity of the two assays was 100%, except for the LiliF^™^ TSUTSU nested PCR kit (98%) (Table 4).

**Table 4:** Comparison of clinical performance between the multiplex SFTSV/OT/IC RT-LAMP assay, PowerChek^™^ SFTSV Real-time PCR kit, and LiliF^™^ TSUTSU nested PCR kit for clinical samples from patients infected with SFTSV or *O*. *tsutsugamushi*.

Clinical samples		Multiplex SFTSV/OT/IC RT-LAMP			PowerChek^™^ SFTSV Real-time PCR kit				LiliF^™^ TSUTSU nested PCR kit
		SFTSV (FAM)	IC (Hex)	OT (Cy5)	M gene (FAM)	S gene (Hex)	M+S gene	IC (Cy5)	
SFTSV (n = 23)	P/N	21/2	22/1	0/23	20/3	22/1	22/1	23/0	-
	Sensitivity	**91.3%**	**95.6%**	**-**	**86.9%**	**95.6%**	**95.6%**	**100%**	**-**
	Specificity	**-**	**-**	**100%**	**-**	**-**	**-**	**-**	**-**
*O*. *tsutsugamushi* (n = 12)	P/N	0/12	8/4	11/1	-	-	-	-	9/3
	Sensitivity	**-**	**66.7%**	**91.6%**	**-**	**-**	**-**	**-**	**75%**
	Specificity	100%	-	**-**	**-**	**-**	**-**	**-**	
Non-infection (n = 100)	P/N	0/100	94/6	0/100	0/100	0/100	0/100	98/2	2/98
	Sensitivity	**-**	**94%**	**-**	**-**	**-**	**-**	**98%**	**-**
	Specificity	**100%**	**-**	**100%**	**100%**	**100%**	**100%**	**-**	**98%**

The sensitivities and specificities were calculated by taking the results of reference SFTSV qRT-PCR and tsutsugamushi qPCR as a standard. P/N: positive/negative ratio

### Cross-reactivity tests of SFTSV/OT/IC RT-LAMP assay with other fever viruses

To confirm the absence of cross-reactivity with other common fever viruses, a total of 38 viral RNA samples, including Hantaan virus, Dengue virus, chikungunya virus, influenza virus A, influenza virus B, RSV A, RSV B, coronavirus 229E, NL63, and OC43, were tested using the multiplex SFTSV/OT/IC RT-LAMP assay (Table 5). As a result, the multiplex SFTSV/OT/IC RT-LAMP assay did not show cross-reactivity for all the tested samples.

**Table 5:** Cross-reactivity of the multiplex SFTSV/*OT*/IC RT-LAMP assay against other fever infectious viruses.

Virus	No	Multiplex SFTSV/OT/IC RT-LAMP		
		SFTSV (FAM)	IC (Hex)	OT (Cy5)
HANV	1	0/1	0/1	0/1
DENV 1–4	4	0/4	3/4	0/4
CHIKV	1	0/1	0/1	0/1
Inf A H1	4	0/4	4/4	0/4
Inf A H3	4	0/4	4/4	0/4
Inf B	4	0/4	4/4	0/4
229E	4	0/4	4/4	0/4
NL63	4	0/4	4/4	0/4
OC43	4	0/4	4/4	0/4
RSV A	4	0/4	4/4	0/4
RSV B	4	0/4	4/4	0/4

HANV: hantaan virus; DENV: dengue virus; CHIKV: chikungunya virus; Inf A H1: Influenza A H1; Inf A H3: Influenza A H3; Inf B: Influenza B; 229E: human coronavirus 229E; NL63: human coronavirus NL63; OC43: human coronavirus OC43; RSV A: respiratory syncytial virus A; RSV B: respiratory syncytial virus B.

## Discussion

Severe fever with thrombocytopenia syndrome virus (SFTSV) and *O*. *tsutsugamushi* are not transmitted by the same vectors; however, these two diseases share a common point as they are transmitted by arthropod bites mostly during outdoor activities [18, 42]. In addition, these two diseases share common clinical features, such as fever, nausea, vomiting, diarrhea, headache, and muscle pain, as well as overlapping disease regions. Furthermore, many cases of co-infection with SFTSV and *O*. *tsutsugamushi* have been reported [43–45].

Here, we developed a multiplex SFTSV/OT/IC RT-LAMP assay to detect SFTSV L segment, groES, and actin beta genes. In sensitivity tests for SFTS and scrub typhus clinical samples, the multiplex SFTSV/OT/IC RT-LAMP assay showed 91.3% and 91.6% for SFTS (n = 23) and scrub typhus (n = 12) clinical samples, respectively. These results are comparable to those (M gene: 86.9% and S gene: 95.6%) of the commercial PowerChek^™^ SFTSV Real-time PCR, and superior to 75% of LiliF^™^ TSUTSU nested PCR kit. The sensitivity and specificity results of PowerChek^™^ SFTSV Real-time PCR were within the scope of previous results (sensitivity: 100% (CI: 73.2%–100%) and sensitivity: 98.1% (CI: 92.5%–99.7%)) reported by Yoo. et al [46]. Therefore, the results of this test are considered reliable, although they were tested with a small number of samples. In addition, the LODs for the multiplex SFTSV/OT/IC RT-LAMP for diluted SFTS and scrub typhus clinical samples were the same as those of the PowerChek^™^ SFTSV Real-time PCR and LiliF^™^ TSUTSU nested PCR kit, respectively.

To simultaneously detect SFTSV and *O*. *tsutsugamushi*, nucleic acid (DNA/RNA) extraction from whole blood is an important step in multiplex SFTSV/OT/IC RT-LAMP assay. Here, Nextractor^®^ NX-48S, which is an automated nucleic acid extraction instrument, was used for fast (within 20 min) and contamination-free extraction of nucleic acids from serum or whole blood. Unfortunately, the multiplex SFTSV/OT/IC RT-LAMP assay could not detect OT signals with nucleic acids (DNA/RNA) extracted from the serum of patients infected with *O*. *tsutsugamushi*. This may be because *O*. *tsutsugamushi* is present at low concentrations in the serum. In fact, most of the *O*. *tsutsugamushi* qPCR and LAMP assays developed to date were performed using DNA extracted from whole blood, buffy coat, and eschars of patients infected with *O*. *tsutsugamushi* [30, 47, 48]. In the sensitivity test, we used 24 SFTS serum clinical samples to confirm the performance of the multiplex SFTSV/OT/IC RT-LAMP assay because of the small number of SFTS whole blood samples. Thus, to confirm whether the multiplex SFTSV/OT/IC RT-LAMP assay can detect SFTSV in whole blood samples, an additional 12 whole blood samples, which were matched with the positive SFTS serum sample, were tested and confirmed to be positive in the multiplex SFTSV/OT/IC RT-LAMP assay (S1 Table).

Our study has some limitations. First, the multiplex SFTSV/OT/IC RT-LAMP assay was performed with a relatively small sample size of positive SFTS (23) and scrub typhus (12) clinical samples, which resulted in widened confidence intervals for our estimates of diagnostic accuracy. However, considering that the multiplex SFTSV/OT/IC RT-LAMP assay showed the same or higher sensitivity compared to the two commercially available diagnostic kits, and 100% specificity to the negative samples, the multiplex SFTSV/OT/IC RT-LAMP assay is sufficiently competitive in commercial development. In addition, it can be applied quickly and cost-effectively, particularly in the early stages of fever in patients in endemic areas. Second, in SFTS LOD test, we used SFTS DNA plasmid as a standard material for LOD test of the multiplex SFTSV/OT/IC RT-LAMP assay. However, there is the deficiency of characterizing an RT-LAMP assay with a DNA standard because it cannot confirm the function of reverse transcriptase. Thus, using SFTS and scrub typhus clinical samples, we reconfirmed the limit of detection of monoplex and multiplex SFTSV/OT/IC RT-LAMP assay compared to those of commercial LiliF^™^ TSUTSU nested PCR and PowerChekTM SFTSV Real-time PCR kit.

In this study, we developed a multiplex SFTSV/OT/IC RT-LAMP assay capable of simultaneous diagnosis of SFTS and scrub typhus within 40 min. For sensitivity and specificity tests with SFTS and scrub typhus clinical samples, the multiplex SFTSV/OT/IC RT-LAMP assay showed similar or superior performance compared with commercial PowerChek^™^ SFTSV Real-time PCR and LiliF^™^ TSUTSU nested PCR. Thus, the multiplex SFTSV/OT/IC RT-LAMP assay could serve as a useful point-of-care molecular diagnostic test for SFTS and scrub typhus.

## Supporting information

S1 Table Sensitivities the multiplex SFTSV/OT/IC RT-LAMP assay for whole blood SFTS clinical samples. (DOCX)

S1 File Raw data. (XLSX)
