# Supplementary material for: Developing a multiplex loop-mediated isothermal amplification assay (LAMP) to determine severe fever with thrombocytopenia syndrome (SFTS) and scrub typhus
Source: PLoS One. 2022 Feb 16;17(2):e0262302. doi: 10.1371/journal.pone.0262302 (PMC8849512; doi:10.1371/journal.pone.0262302)
Supplement: S1 Table — (DOCX) [file pone.0262302.s001.docx]

**S1 Table. Sensitivities the multiplex SFTSV/OT/IC RT-LAMP assay for whole blood SFTS clinical samples.**

| **Sample No** | **Multiplex SFTSV/OT/IC RT-LAMP** | | | | | |
| --- | --- | --- | --- | --- | --- | --- |
|  | **FAM (SFTS)** | | **CY5 (TSUTSU)** | | **HEX (IC)** | |
|  | **CT** | **RFU** | **CT** | **RFU** | **CT** | **RFU** |
| 1 | 14.44 | 10980 | N/A | 257 | N/A | 476 |
| 2 | 19.04 | 14899 | N/A | 196 | N/A | 716 |
| 3 | 20.28 | 13502 | N/A | 294 | N/A | 536 |
| 4 | 13.23 | 13049 | N/A | -12.2 | N/A | 641 |
| 5 | 12.13 | 14632 | N/A | 450 | N/A | 681 |
| 6 | 16.06 | 11942 | N/A | -3.83 | N/A | 675 |
| 7 | 23.75 | 12060 | N/A | 234 | N/A | 565 |
| 8 | 21.44 | 13790 | N/A | 61.5 | N/A | 704 |
| 9 | 12.38 | 13678 | N/A | 512 | N/A | 550 |
| 10 | 13.11 | 13025 | N/A | -16 | N/A | 581 |
| 11 | 16.52 | 13439 | N/A | 489 | N/A | 564 |
| 12 | 21.99 | 11889 | N/A | 70 | N/A | 390 |
